# ssDNA Pairing Accuracy Increases When Abasic Sites Divide Nucleotides into Small Groups

**DOI:** 10.1371/journal.pone.0130875

**Published:** 2015-06-26

**Authors:** Alexandra Peacock-Villada, Vincent Coljee, Claudia Danilowicz, Mara Prentiss

**Affiliations:** Department of Physics, Harvard University, 17 Oxford St., Cambridge, MA 02138, United States of America; University of North Carolina at Charlotte, UNITED STATES

## Abstract

Accurate sequence dependent pairing of single-stranded DNA (ssDNA) molecules plays an important role in gene chips, DNA origami, and polymerase chain reactions. In many assays accurate pairing depends on mismatched sequences melting at lower temperatures than matched sequences; however, for sequences longer than ~10 nucleotides, single mismatches and correct matches have melting temperature differences of less than 3°C. We demonstrate that appropriately grouping of 35 bases in ssDNA using abasic sites increases the difference between the melting temperature of correct bases and the melting temperature of mismatched base pairings. Importantly, in the presence of appropriately spaced abasic sites mismatches near one end of a long dsDNA destabilize the annealing at the other end much more effectively than in systems without the abasic sites, suggesting that the dsDNA melts more uniformly in the presence of appropriately spaced abasic sites. In sum, the presence of appropriately spaced abasic sites allows temperature to more accurately discriminate correct base pairings from incorrect ones.

## Introduction

Several assays depend on the design of single-stranded DNA (ssDNA) molecules that will accurately bind to their complementary ssDNA strands [1–3]. In these assays the discrimination between fully complementary sequences and those that contain one or more mismatches depends on the difference between the corresponding melting temperatures. During melting, or thermal denaturation, two single strands become separated due to an increase in temperature [4]. At low temperatures only partial separation takes place till full melting is achieved above the melting temperature, T_m_ [5]. For matched sequences shorter than ~ 20 base pairs (bp) the T_m_ increases significantly with N [6]; however, T_m_ eventually approaches an asymptotic value [7]. For ~ 35-bp sequences, the presence of mismatches may not change the melting temperature significantly, allowing incorrect sequences to remain bound for long times at temperatures where correctly matched pairings are barely stable. Such kinetic trapping can be reduced by using buffers that weaken base pairing [8,9]. Unfortunately, weakened base pairing makes stringency more challenging since it also decreases the free energy difference between correctly and incorrectly paired bases.

Part of the problem with temperature based mismatch detection is that long dsDNA does not melt uniformly; instead, regions with weaker base pairing melt at lower temperatures than regions with stronger base pairing. The melting temperature change associated with a mismatch depends not only on the nature of the mismatch, but also on the composition of nearby bases, as well as the sequence distribution along the entire dsDNA.

Given that some mismatches have melting temperatures that are nearly indistinguishable from correct pairings, development of commercial products that use temperature to accurately detect mismatches require significant investments of time and money to create systems that make the recognition of a particular target much more accurate than the output of individual sequence comparisons. In this work we show that accuracy of individual sequence comparisons can be greatly improved by using probes with appropriately spaced abasic sites in between groups of bases. This improvement in the accuracy of individual sequence comparisons may allow faster and more inexpensive development of assays for sequences for which there are no existing tests.

## Materials and Methods

### Sample Preparation

Oligodeoxynucleotides (oligos) were synthesized using phosphoramidite chemistry (Integrated DNA Technologies, IDT) and purified by high-performance liquid chromatography. Stable abasic sites in all of the oligonucleotides were introduced using 1′,2′-Dideoxyribose modification (IDT). Each oligo was dissolved in molecular biology grade distilled water at 100 μM. Texas Red and BHQ-1 were used as a fluorophore-quencher couple having been identified as a thermally stable combination providing close to equal fluorescence /quenching over a range from 25 to 90°C while the melting data derived using this couple most closely resembled that of data observed by melting oligonucleotides without attached fluorophores [10]. Measurements of fluorophore and quencher couples were performed with 1 μM solutions of each oligonucleotide in 10 mM Tris pH = 8.0 and at the NaCl concentrations indicated for each experiment.

### Fluorescence

Full fluorescent spectra of the Texas Red fluorophore oligonucleotides and BHQ-1 quencher oligonucleotides were taken from 400 to 700 nm to establish a baseline. Fluorescence readings for each 500 μl sample were measured at an excitation wavelength of 596 nm and emission wavelength of 611 nm with 1-nm slit widths every 1°C/min using a FluoroMax-4 spectrofluorometer (Horiba). One data point was taken at each temperature. Both increasing and decreasing temperature cycles were taken. The melting temperature was determined using two different methods. In one method, Tm_deriv_ is the temperature at which the derivative of the fluorescence versus temperature is a maximum. That method eliminates systematic errors associated with the inaccurate estimation of the minimal fluorescence. In addition, this technique removes effects associated with constant variations in fluorophore output with temperature since such a slope produces a constant offset in the derivative that does not affect the position of the derivative maximum. In the other method, Tm_1/2_ is the temperature where 50% of the molecules that annealed have melted. The results are very similar, as shown in Table 1, indicating that systematic errors that vary between the two techniques did not affect significantly the measured melting temperatures.

**Table 1:** Measured T_m_ values found using R experiments on systems without any abasic sites (columns 2–5) and the system with 7 abasic sites dividing the system into 6 groups of 4 bases (columns 6–9).

MP	Ave Tm_1/2_ without abasic sites (°C)	ΔTm_1/2_ without abasic sites (°C)	Ave Tm_deriv_ without abasic sites (°C)	ΔTm_deriv_ without abasic sites (°C)	Ave Tm_1/2_ with abasic sites (°C)	ΔTm_1/2_ with abasic sites (°C)	Ave Tm_deriv_ with abasic sites (°C)	ΔTm_deriv_ without abasic sites (°C)
0	79.75 ±1	NA	81.5±1	NA	26.5±1	NA	27.5±1	NA
6	73.75±1*	6 ±1.4*	76±1*	5.5±1.4*	21.25±1	5.25±1.4	23±1	4.5±1.4
15	76.25±1	3.5 ±1.4	79±1	2.5±1.4	17.25±1	9.25±1.4	20±1	7.5±1.4
17	73.75±1	6 ±1.4	76±1	5.5±1.4	19±1	7.5±1.4	20±1	7.5±1.4
18	76±1	3.75±1.4	79±1	2.5±1.4	NA	NA	NA	NA
19	76.25±1	3.5±1.4	78.5±1	3±1.4	17.25±1	9.25±1.4	17.5±1	10±1.4
21	77±1	2.75±1.4	79±1	2.5±1.4	17.5±1	9±1.4	18±1	9.5±1.4
30	77.25±1	2.5±1.4	78.75±1	2.75±1.4	17.75±1	8.75±1.4	19.5±1	8±1.4

## Results and Discussion

**Fig 1:**
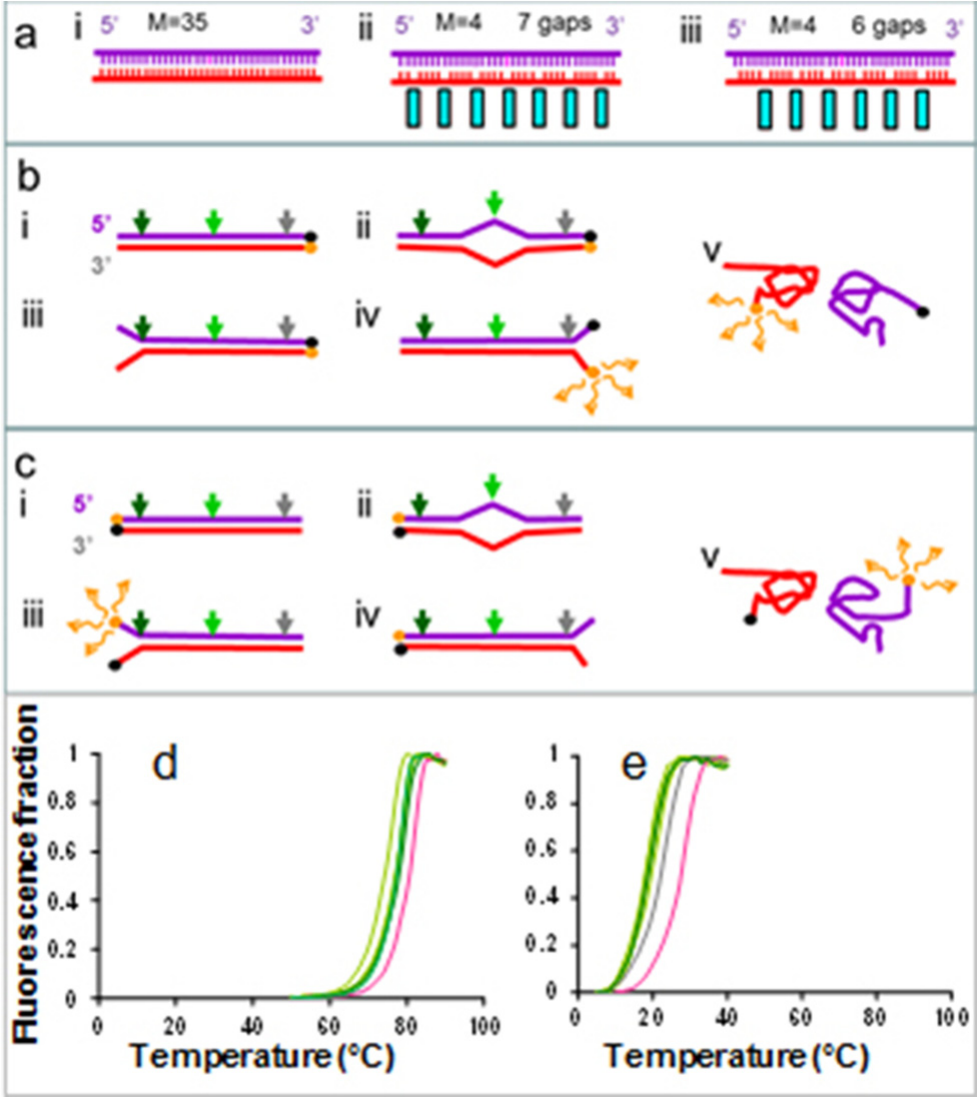
Measurements of the melting temperature, T_m_. **(a)** Schematic of the pairing of the target ssDNA containing 35 nucleotides (purple) with the ssDNA probe (red), (i) probe with no abasic sites, (ii) and (iii) probes divided by abasic sites into groups with size M. i. M = 4 nt groups. The positions of the abasic sites are highlighted by the cyan rectangles. ii and iii correspond to the same M = 4 value, but have different numbers of abasic sites because they are shifted with respect to each other by 2 bases. **(b)** Schematic of the melting experiment with R constructs. **(c)** Schematic of the melting experiment with L constructs. The colored arrows indicate positions of different single isolated mismatches. The black and orange circles indicate the positions of the BHQ-1 and Texas Red fluorophores. Fluorescence is high if the fluorophores are widely separated, but low if they are close. (ii)-(iv) show partial melting and (v) shows full melting. **(d)** Texas Red fluorescence as a function of temperature in a buffer containing 150 mM NaCl for the system shown in a-i; perfectly matched sequence (magenta) and different single mismatches (green and gray as specified in Fig 3). **(e)** Texas Red fluorescence as a function of temperature in a buffer containing 150 mM NaCl for the system shown in a-ii and same color code as in (d).

Fig 1A shows a schematic of some ssDNA pairings used in this work (S1 Table) where each system consisted of target ssDNA molecules containing no abasic sites (purple). These ssDNA targets were allowed to pair with ssDNA probes containing various numbers of abasic sites (red). Fig 1A–i shows a probe containing no abasic sites. The cyan bars in Fig 1A-ii and 1A-iii highlight the location of the abasic sites which disrupt the stacking between neighboring groups of nucleotides. The molecule in Fig 1A-ii contains 7 abasic sites whereas in Fig 1A-iii has only 6; however, in both cases the bases are divided into groups of 4 which are separated by abasic sites.

Fig 1B and 1C illustrates the experimental schematic for the detection of pairing using a fluorophore/quencher couple consisting of a Texas Red fluorophore and a BHQ-1 quencher. If the two ssDNA molecules are completely paired, the fluorescence signal from the Texas Red fluorophore at the end of one ssDNA will be strongly suppressed due to the proximity of the BHQ-1 quencher on the corresponding end of the other ssDNA. If the two ssDNA molecules are completely separated the fluorescence due to the Texas Red fluorophore will be strong.

Of course assays that rely on fluorophores at the ends of molecules really only detect melting at those molecules’ ends. Since a long dsDNA molecule does not usually melt uniformly, it is quite possible that there are temperatures at which one end of the molecule will be melted, while other parts of the molecule remain bound. In these cases, the fluorophore pair at the melted end would not provide accurate information on the complete separation of the two ssDNA molecules. In order to reduce misreporting due to the position of the fluorophore, for each base pairing we conducted two different sets of experiments. In one set of experiments the fluorophore pair was attached to one end of the dsDNA, and in the other set of experiments the fluorophore pair was attached to the other end of the dsDNA. We designated the two types of experiments as R and L measurements, respectively. Fig 1B and 1C shows the schematic for the R (L) measurements where the BHQ-1 molecule is on the 3´ end of the target (probe) ssDNA, while the Texas Red fluorophore is on the 5´ end of the probe (target) molecule. The sequences for all of the R and L experiments are available in SI, where each base pairing is illustrated, and the positions of the abasic sites, mismatches, and fluorophores are highlighted.

As Fig 1B and 1C illustrate, the fluorophores can report melting at an end even if other portions of the dsDNA remain bound. In this case, the melting temperature reported by the fluorophore pair nearest the mismatch will be lower than the melting temperature reported by the pair that is more distant from the mismatch. For mismatches near the ends of the molecules, this effect is clearly seen in dsDNA that does not contain abasic sites. In particular, mismatches for sequences with mismatches at position 8 or at position 30, the fluorophore pair nearest the mismatch reports a lower melting temperature than the fluorophore pair more distant from the mismatch; however, in what follows we will show that for the system that is divided by basic sites into 4 bp groups, the two fluorophore pairs report indistinguishable melting temperatures indicating that the dsDNA melts along the entire length instead of melting locally while preserving base pairing in some other region of the sequence.

Fig 1D and 1E shows the normalized fluorescence intensity as a function of increasing temperature for R measurements. Fig 1D shows the results for pairing with no abasic sites, which was illustrated in Fig 1A-i. Fig 1E shows the results for the pairing illustrated in Fig 1A-ii. The magenta lines indicate the curves for the case where the sequences are perfectly matched. The other colors represent results for the different single mismatches. Fig 1D and 1E clearly show that the T_m_ values are much higher for the undivided 35 bases than they are for pairings where the probe ssDNA is divided into groups of 4 by abasic sites.

**Fig 2:**
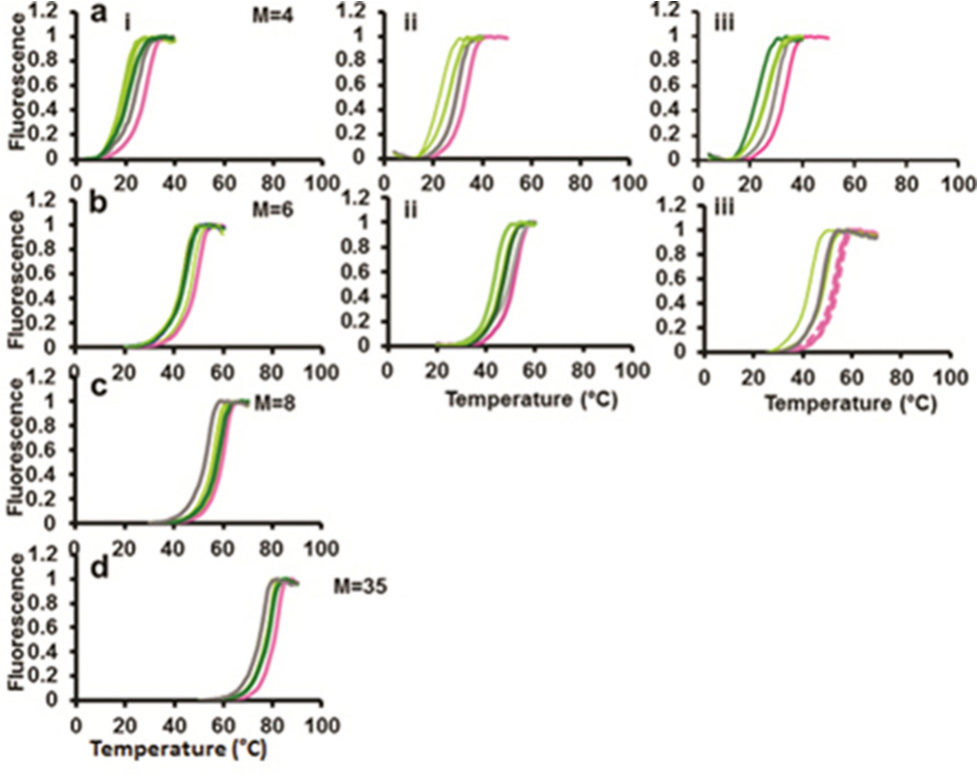
Measurements of the melting temperature, T_m_, for sequences divided by abasic sites into groups with size M for the same experimental conditions used to obtain the data in Fig 1. **(a)** M = 4 results where i is the same as Fig 1D, ii is the L result for the same system, and iii is the R result for the system shown in main text Fig 1A-iii where the abasic sites are shifted by two with respect to the system shown in i and ii. **(b)** Analogous results for a system with M = 6. For the M = 6 system that is shifted, some single mismatches fall on an abasic site. Curves corresponding to mismatches on abasic sites are shown as magenta dashed lines. The results are in very good agreement with the perfect match. **(c)** Analogous results for a system with M = 8. **(d)** Analogous result for an undivided probe.

Though the data in Fig 1 indicates that including abasic sites has a strong effect on the melting behavior of dsDNA, it is not clear whether the result is due to the mere presence of abasic sites, or whether the grouping of the abasic sites also plays an important role. In Fig 1, the sequences are divided into groups of M = 4, separated by abasic sites. Fig 2 shows melting curves for larger M values. The results in Fig 2 clearly indicate that the melting temperatures increase as the group size increases. This is not surprising since the presence of abasic sites must alter stacking interactions. Interestingly, the melting curves for matched base pairings (magenta) are indistinguishable from the melting curves for pairings in which the “mismatches” fall on abasic sites (dashed magenta curves). In addition, the melting curves for the two M = 4 systems are very similar even though the position and number of the abasic sites are different for the two cases. Thus, positions of individual mismatched bases could be detected by sliding the position of the abasic sites while maintaining group size.

We used fluorescence vs. temperature curves to determine the melting temperatures, T_m_, for different base pairings. The curves shown in Fig 1 were obtained by increasing the temperature as a function of time. We obtained melting temperature information by cycling the temperature up and down. The up and down curves show a systematic shift that depends on the rate at which the temperature was cycled. We checked that the average of the two curves did not shift as a function of cycling rate. Table 1 provides a summary of experiments associated with the graphs shown in Fig 1. Table 1 includes melting temperature measurements based on the measuring of the temperature at which the derivative of the fluorescence curves is a maximum (Tm_deriv_), as well as measurements based on the temperature at which 50% of the pairing has melted (Tm_1/2_). As the table shows, the results for both techniques are very similar. Since systematic errors in the two techniques differ, this suggests that both techniques are providing accurate reporting to within the reported error.

The numbers shown are the averages (Ave) temperatures in degrees Celsius for up and down curves. The first column is the position of the mismatch, MP. The second and sixth columns show the results for T_m1/2_ measurements that use the half fluorescence point to estimate T_m_. The fourth and eighth columns show the results for Tm_deriv_ measurements that use temperature at which the derivative of the fluorescence with respect to temperature is a maximum. The third, fifth, seventh, and ninth columns show the differences between the T_m_ value for the perfect match and the T_m_ value of the corresponding mismatch. The asterisks indicate values where the R measurements indicated a significantly lower melting temperature than the L experiments, suggesting that the R measurements are invalid.

**Fig 3:**
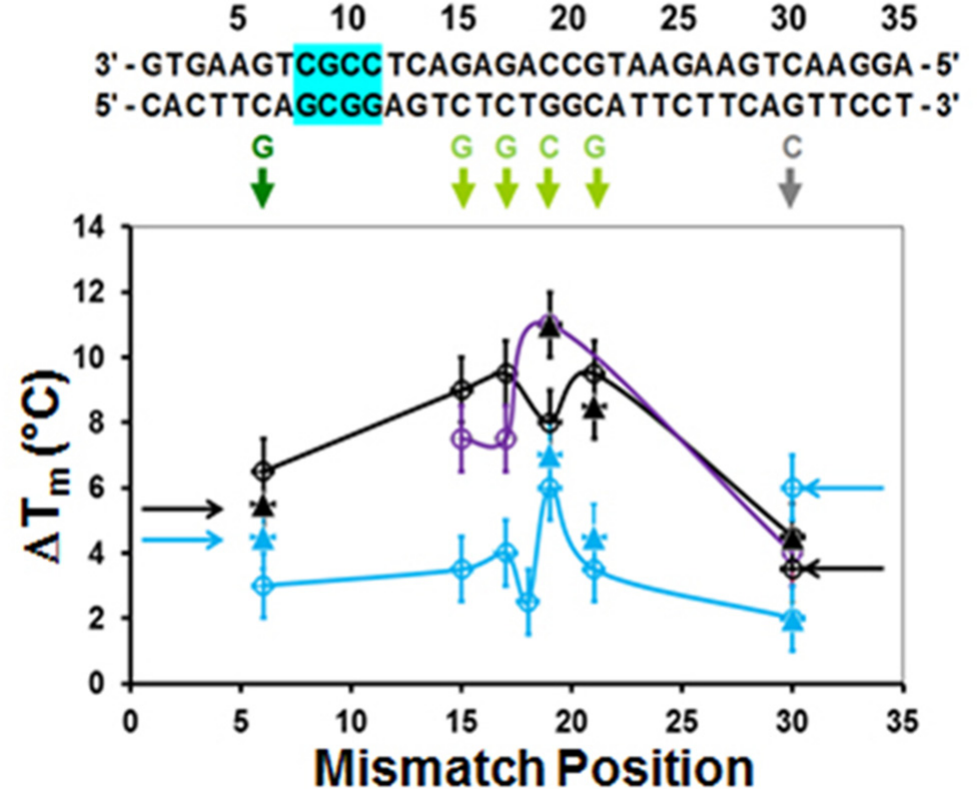
ΔT_m_ as a function of the position of single mismatches. The sequence is shown at the top of the figure The single base pair mismatch replacements are shown below in colors corresponding to the colors of the curves shown in Fig 1D and 1E. The cyan, black, and purple lines and symbols correspond to the systems shown in Fig 1i, ii, and iii, respectively. The solid triangles represent the L data. The hollow circles correspond to the R data. The solid lines connect the data points from R measurements, except for the T_**m**_ mismatch at position 30, which is derived from the L data. The arrows on the right (left) side of the graph indicate T_**m**_ values calculated from R (L) measurements for a mismatch at position 30(8). For the undivided probe, the fluorophore pair nearest the mismatch separates at a significantly lower temperature than the true T_**m**_, but for the divided probe the ends melt at the true T_**m**_.

Fig 1E shows fluorescence curves for a 35-nt sequence containing 7 abasic sites that separate the nucleotides into groups of 4. Of course, one could distribute the abasic sites differently, so that the nucleotides were divided into larger or smaller groups. Fig 2 shows results analogous to those shown in Fig 1E, except that the results correspond to systems with different distributions of abasic sites. The M values shown in the Fig correspond to the number of nucleotides that occupy the regions between abasic sites. Fig 2Ai is the same as Fig 1E. It corresponds to the R experiment results for the M = 4 case that includes 7 abasic sites. The corresponding sequences are shown in SI under the heading “6 groups with 4 bp each = 7 spacers”. Fig 2Aii shows the analogous results for the L experiments where the fluorophores are on the end opposite to the end used in the R experiments. Fig 2Aiii is the result for R experiments on a system with M = 4, but only 6 abasic sites. The corresponding sequences are shown in SI under the heading “7 groups with 4 bp each = 6 spacers”. All three sets of results are very similar, as Fig 3 illustrates. Thus, T_m_ measurements are not distorted by using fluorophore pairs at the ends of the molecules, and what is relevant is not the number of abasic sites, but the size of the nucleotide groups into which the abasic sites are divided.

In order to determine the effects of single base pair mismatches on T_m_, we calculated ΔT_m_, the difference between the T_m_ for the perfect match and the T_m_ for pairing with different single base pair mismatches. The sequence at the top of Fig 3 is the original target sequence with which the various probes are paired. The single base replacements in the mismatched targets are indicated by the colored letters directly below the original target sequence. The cyan rectangle indicates the GC-rich clamp which is the most stably bound region. The color of the curves in Fig 1D and 1E corresponds to the color of the letters and colored arrows highlighting the corresponding mismatches in Fig 3 The results for L measurements are shown in Fig 2Aii.

Fig 3 shows graphs of ΔT_m_ as a function of mismatch position for the three pairings illustrated in Fig 1A-i (cyan), ii (black), and iii (purple). The hollow circles and the solid triangles correspond to the T_m_ values determined by the R and L measurements, respectively. The lines connect the T_m_ values for R measurements, with the exception of the mismatch at position 30 which is taken from the L measurements to avoid artifacts associated with the melting of the ends.

Fig 3 shows that the probes illustrated in Fig 1A-ii and 1A-iii have similar ΔT_m_ values even though they have different numbers of abasic sites which are located in different positions, suggesting that it is the group size that is important rather than the position of the abasic sites. In addition, the similarity between the results for the two systems containing different numbers of abasic sites shows that the position of the mismatch within a 4-bp group does not have a significant effect on ΔT_m_. Furthermore, the ΔT_m_ values for the systems containing the abasic sites are consistently larger than those for the undivided 35-nt probe, and the standard deviation values between the ΔT_m_ values are smaller for the system containing the abasic sites than for the undivided 35-nt probe. Importantly, for the system with the abasic sites variations for mismatches near the center of the molecule are much smaller than the variation for the undivided probe. Finally, for the system divided by abasic sites, the half fluorescence temperatures for the mismatches nearest the fluorophores are the same for both the R and L measurements indicating that the entire molecule melts at almost the same temperature, whereas the results for the undivided 35-bp DNA show that the ends open at a temperature well below T_m_.

**Fig 4:**
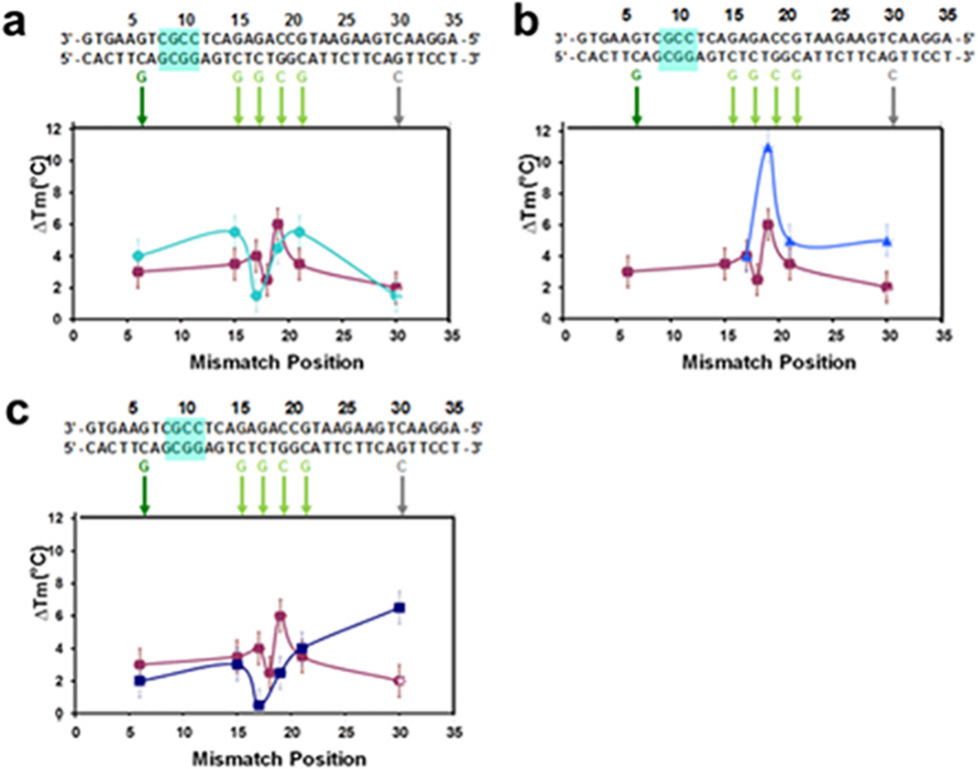
Effect of M on melting curves: differences between the ΔT_m_ for a perfectly matched 35 bp sequence with and without abasic sites and various single bp mismatches in 150 mM NaCl. The sequence is shown in black at the top of the figure. The mismatches are shown below the sequence. In all graphs, the purple squares correspond to a 35 bp sequence without abasic sites for the R data except for the mismatch at position 30 which was taken from the L data. **(a)** Results for 35 bp sequence with 5 abasic sites where M = 6 (aqua circles). **(b)** Results for another case where M = 6 shifted by two base pairs from the system shown in (a) (blue triangles). **(c)** Results for 35-bp sequences with 3 abasic sites where M = 8 (dark blue squares).

Analogous results for probes divided into larger groups are shown in Fig 4. That figure shows that the insertion of abasic sites does not enhance mismatch detection if the spacing between the basic sites is too large. The top row corresponds to results for M = 6, where Fig 4A and 4B correspond to the melting curve data shown in Fig 2Bi and 2Biii, respectively. The results in Fig 4A show no significant improvement over results in the absence of abasic sites, and the results shown in Fig 4B are somewhat worse than the undivided result. The results in Fig 4C correspond to the M = 8 results shown in Fig 2C. Like the M = 6 results, the M = 8 results are not an improvement over the undivided system. For the M = 8 results the high ΔT_m_ is an artifact due to partial melting near the fluorophore, indicating that by M = 8 the melting along the length of the dsDNA is no more uniform than it was for the case without abasic sites. Thus, the data shown in Fig 4 highlights the importance of dividing the nucleotides into small groups.

So far we have considered the melting temperatures for different annealing experiments. We also investigated a base pairing assay based on the change in fluorescence with temperature that oscillates in the temperature region where the mismatches have melted out, but the correct match continues to show some base pairing. This technique offers an oscillating fluorescence signal that can increase the accuracy of the discrimination between matched and mismatched pairings. Heterodyne detection can be used to increase the signal to noise in such experiments. We performed these experiments for base pairings whose fluorescence curves are shown in Fig 1D and 1E. We compared the results for the mismatches that were least detectable in each system. For the abasic system shown in Fig 1Aii, this is the mismatch at position 30 for the M = 4 abasic, whereas for the system without abasic sites the mismatch at position 6 is the hardest to detect. Experiments on the annealing in the absence of abasic sites show that oscillating the temperature between 83 and 87°C produces a fluorescence that oscillates between 100% of peak fluorescence and 98% of peak fluorescence in the base pairing with a mismatch at position 30, whereas the perfect match oscillates between 100% and 92% of peak fluorescence. The difference in the oscillation amplitude for the correct match and the nearest mismatch was ~ 6%. In contrast, the system with M = 4 with a mismatch at position 6, oscillating between 41 and 36°C produces fluorescence values oscillating between 100% and 99% of peak fluorescence, whereas the perfect match oscillates between 100% and 76% of peak fluorescence. The difference in the oscillation amplitude for the correct match and the nearest mismatch was ~ 23%. Thus, the fluorescence difference in the abasic system is more than 3x larger than the difference in the system without abasic sites.

As demonstrated in this paper, grouping the nucleotides in the ssDNA probe into 4 nt groups separated by abasic sites can greatly improve mismatch detection by making the ΔT_m_ values for different mismatches larger and more consistent than the values for undivided probes. We propose that separating the bases in the ssDNA into groups of 4 nucleotides using abasic sites has the following effects: 1. It significantly reduces the melting temperatures. 2. It makes samples melt more uniformly because no clamp can extend more than 4 bp whereas an undivided system can have much longer clamps which may remain paired at temperatures where mismatches have destabilized pairing in other regions of the sequence. 3. It allows a single mismatch to strongly destabilize an entire group since any single mismatch and its neighbors make up at least half of the nucleotides within a group. The last suggestion is consistent with experimental results that showed that a single base can significantly destabilize a sequence with a length of up to ~ 7 nt [11], so one would expect discrimination to improve for groups containing fewer than 7 bp. We propose that this explains why ΔT_m_ is insensitive to the position of the mismatch within an individual 4-base pair group as well as the position within the 35 nt sequence.

Though a mismatch cannot be distinguished if the mismatch falls on an abasic site, a base pairing assay could contain sequences where the abasic sites are shifted by 2 bases, so such mismatches could still be readily detected. Similarly, in a 35-nt system including targets shifted by 17 nt would move mismatches that were near the ends to positions near the middle of the sequence where systems using 4 nt probes readily detect them. This shift is much less helpful in systems without abasic sites because even mismatches near the middle show a large variation in ΔT_m_. Systems that work at lower temperatures and are less sensitive to temperature should be easier to build and less expensive. Thus, the incorporation of abasic site in probe sequences may make the development of new base pairing assays faster and less expensive, increasing the range of applications in which base pairing assays are commercially viable.

## Supporting Information

S1 Table Sequences used in this work. The table shows the sequences used in the R and L pairing experiments.
(DOCX)
