# Supplementary material for: ssDNA Pairing Accuracy Increases When Abasic Sites Divide Nucleotides into Small Groups
Source: PLoS One. 2015 Jun 26;10(6):e0130875. doi: 10.1371/journal.pone.0130875 (PMC4482597; doi:10.1371/journal.pone.0130875)
Supplement: S1 Table — The table shows the sequences used in the R and L pairing experiments. (DOCX) [file pone.0130875.s001.docx]

**ssDNA pairing accuracy increases when abasic sites divide nucleotides into small groups**

Alexandra Peacock-Villada, Vincent Coljee, Claudia Danilowicz, Mara Prentiss

**Supporting Information**

**S1 Table.** Sequences used in this work.

The table shows the sequences used in the R and L pairing experiments.
